# Molecular driver alterations and their clinical relevance in cancer of unknown primary site

**DOI:** 10.18632/oncotarget.10035

**Published:** 2016-06-14

**Authors:** Harald Löffler, Nicole Pfarr, Mark Kriegsmann, Volker Endris, Thomas Hielscher, Philipp Lohneis, Gunnar Folprecht, Albrecht Stenzinger, Manfred Dietel, Wilko Weichert, Alwin Krämer

**Affiliations:** ^1^ Clinical Cooperation Unit Molecular Hematology/Oncology, German Cancer Research Center (DKFZ) and Department of Medicine V, University of Heidelberg, Heidelberg, Germany; ^2^ Institute of Pathology, University of Heidelberg, Heidelberg, Germany; ^3^ Institute of Pathology, Technische Universität München, Munich, Germany; ^4^ Division of Biostatistics, German Cancer Research Center (DKFZ), Heidelberg, Germany; ^5^ Institute of Pathology, Charité-Universitätsmedizin Berlin, Berlin, Germany; ^6^ Medical Department I, University Hospital Carl Gustav Carus, Dresden, Germany; ^7^ Department of Pathology, Center for Integrated Diagnostics (CID), Massachusetts General Hospital/Harvard Medical School, Boston, USA; ^8^ National Center of Tumor Diseases (NCT), Heidelberg, Germany; ^9^ Member of The German Cancer Consortium (DKTK), Heidelberg, Germany

**Keywords:** CUP, carcinoma of unknown primary, massive parallel sequencing, driver mutations, p53

## Abstract

Cancer of unknown primary (CUP) is defined as metastatic solid malignancy where no primary tumor is detected despite appropriate staging. About 90% of CUP represent adenocarcinoma or undifferentiated carcinoma. Since therapy regimens are only modestly effective, identification of the molecular landscape of these neoplasms might be a promising approach to direct CUP therapy and aid in tumor classification. We screened a cohort of 128 patients with adenocarcinoma or undifferentiated carcinoma meeting the definition of CUP. Massive parallel multigene sequencing of 50 genes, which had been selected due to their relevance as oncogenic drivers or druggable molecular targets could ultimately be performed on samples from 55 patients for whom complete clinical datasets were also available. Overall, 60 tumor-specific mutations and 29 amplifications/deletions, as revealed by coverage analysis, were detected in 46 cases (84%). The most frequently mutated genes were *TP53* (30 cases, 55%), *KRAS* (9 cases, 16%), *CDKN2A* (5 cases, 9%), and *SMAD4* (5 cases, 9%). The most frequently deleted gene was CDKN2A (8 cases, 15%). *KRAS* and *CDKN2A* mutations significantly correlated with poor progression-free survival (PFS) and, in case of *KRAS*, overall survival (OS). WIldtype *TP53* and female sex defined a relatively favorable category, with favorable PFS and OS. 8 cases (15%) harbored mutations that may be targetable by currently approved drugs. Taken together, Mutations of relevant driver genes are present in the vast majority of CUP tumors. Some of them impact on prognosis and a subset is putatively druggable.

## INTRODUCTION

Cancer of unknown primary (CUP) refers to solid malignancies where no primary tumor is detected despite appropriate staging [1–3]. 50–70% of CUP cases are adenocarcinomas and 20–30% undifferentiated carcinomas. Squamous cell carcinomas, neuroendocrine carcinomas and other rare malignancies together account for the remaining 10% of CUP cases [1–4]. By combining traditional histologic, immunohistochemical and clinical criteria, 10–30% of CUP patients can be assigned to prognostically favorable subgroups, which include patients that benefit from management in analogy to specified organ cancers and cases with locally restricted disease warranting curative approaches [2, 4, 5]. The prognosis of the remaining majority of patients is dismal. The standard approach in these cases is to offer non-specific cytostatic therapy in palliative intention [2, 3, 6].

In an increasing number of tumor entities, drugs designed to target molecular alterations have been successfully implemented into therapeutic strategies. Ideally, these targeted therapies should be applied as guided by molecular predictors, e.g. activating *EGFR* mutations triggering therapy by drugs targeting EGFR [7–9]. In CUP, targeted therapies are not established, which is explained by the lack of data on molecular alterations in this entity. The majority of publications on mutations and other aberrations of potential oncogenic drivers in CUP date back to the era before high-throughput sequencing and suffer from small sample sizes and heterogeneous methodology [10–12]. Only lately, two studies on next-generation sequencing of CUP samples were published [13, 14].

In the present study, we characterized molecular aberrations in CUP cases belonging to the most relevant histological categories of adeno- or undifferentiated carcinomas using a panel of 50 genes selected according to their relevance as potential therapeutic targets or biologically important oncogenic drivers. Moreover, detailed clinical data including progression-free survival (PFS) and overall survival (OS) were available, thereby enabling in-depth analyses of relevant clinical correlations.

## RESULTS

### Patient characteristics

A total of 128 CUP patients with adeno- or undifferentiated carcinoma meeting the eligibility criteria were identified. After exclusion of cases due to insufficient tissue or DNA quality, withdrawal of consent, or revision of diagnosis, the final study population consisted of 55 successfully sequenced cases with complete clinical datasets (Supplementary Figure 1). The high dropout rate due to lack of representative tissue material is explained by the fact that in CUP, the histological diagnosis is usually established on core needle biopsies which have to be subjected to extensive immunophenotyping to exclude organ-specific differentiation. The median follow-up of patients was 28.9 months. During follow-up, 39 deaths and 38 disease progressions were observed. OS was 7 months for male patients and 17 months for female patients (*P* = 0.14). Patient characteristics are summarized in Table 1.

**Table 1:** Patient characteristics (*n* = 55)

Age at diagnosis	59 ± 10 years^*^	
**Gender**		
Females	28 cases	(51%)
Males	27 cases	(49%)
**Affected organ sites**		
Lymph nodes	33 cases	(60%)
Liver	17 cases	(31%)
Bones	14 cases	(25%)
Lung	11 cases	(20%)
Peritoneum	10 cases	(18%)
Adrenals	8 cases	(15%)
Brain	2 cases	(4%)
Skin	2 cases	(4%)
Others organ sites	7 cases	(13%)
**Number of affected organ sites**		
1	24 cases	(44%)
2	15 cases	(28%)
3	11 cases	(20%)
4	2 cases	(4%)
5	2 cases	(4%)
Mean	1.9 ± 1.1^*^	
**Median progression-free survival**	7.0 (5.0–9.0) months^†^	
**Median overall survival**	16.2 (7.6–23.4) months^†^	

Annotations:

* mean ± standard deviation.

† median with 95% confidence interval.

### Molecular alterations in CUP patients

**Figure 1:**
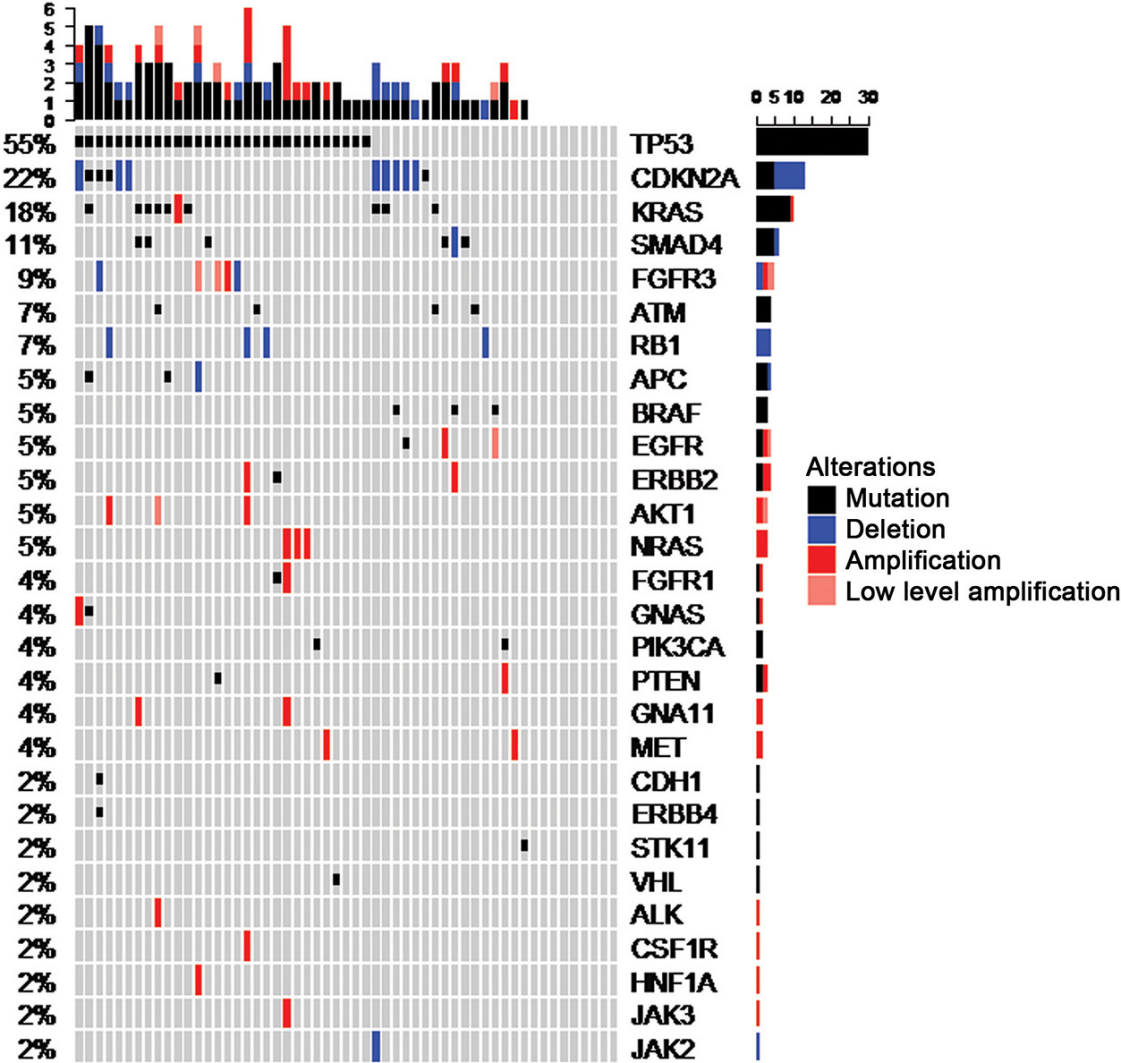
OncoPrint summarizing mutations and copy number alterations of the 55 CUP cases included in the final dataset

Panel sequencing of 50 selected genes (Supplementary Table 1) revealed at least one molecular alteration in 46 out of 55 cases (84%), including mutations in 43 cases (78%) and CNVs (amplifications/deletions), as revealed by coverage analysis, in 29 cases (53%). In total, 60 tumor-specific mutations and 29 CNVs were detected (Supplementary Table 2). Individual cases harbored up to 5 mutated genes, 4 amplified genes, 2 deleted genes, or 6 molecular alterations in total (Figure 1). The gene most frequently affected was *TP53*, of which 33 mutations were detected in 30 cases (55%), including three cases with two mutations per case. *CDKN2A* was affected in 12 cases (22%) including 8 deletions (15%) and 5 mutations (9%). One case showed a *CDKN2A* deletion together with a *CDKN2A* mutation. *KRAS* was affected in 10 cases (18%) including 9 mutations (16%) and one amplification (2%). The 2 detected *EGFR* mutations were located within exons 18 and 21, compatible with activating mutations [9], and 2 out of 3 *BRAF* mutations coded for the V600E mutant protein [15]. In both instances, these are established molecular predictors for approved targeted therapies in some entities.

### Correlation of molecular alterations to clinical data

The mutational status of the most frequently altered genes, *TP53*, *CDKN2A*, *KRAS* and *SMAD4*, was tested for correlations to survival data (Table 2). Mutations of *KRAS* and *CDKN2A* significantly correlated to poor PFS, with *KRAS* mutations also showing a significant correlation to poor OS. *TP53* mutations were associated with a slightly shorter OS, but this association was not significant based on the log-rank test. Applying a test also able to detect crossing hazard rates [16] revealed a *P* value of 0.055, indicating the possibility that TP53 mutations may confer an increased risk of early death. Moreover, the mutational status of *TP53* interacted with the prognostic impact of gender (Supplementary Figure 2): A significantly better OS (*P* = 0.003) and PFS (*P* = 0.007) of female versus male patients was only found in cases lacking *TP53* mutations (PFS interaction *P* =0.08, OS interaction *P* = 0.27).

**Table 2:** Correlations of frequent molecular alterations to survival data

Molecular alteration		Overall survival^*^	Progression–free survival^*^
*TP53* mutation	present (*n*= 30)	10.7 (6.0–NA) months	7.0 (4.1–13.0) months
	absent (*n* = 25)	17.4 (7.6–27.0) months	7.4 (4.2–12.1) months
	*P*^†^	0.85	0.97
*KRAS* mutation	present (*n*= 9)	6.0 (4.9–NA) months	3.3 (2.0–NA) months
	absent (*n* = 46)	17.4 (8.7–25.4) months	7.4 (5.9–12.1) months
	*P*^†^	**0.016**	**0.005**
*CDKN2A* deletion	present (*n*= 8)	15.4 (7.1–NA) months	7.9 (3.0–NA) months
	absent (*n* = 47)	16.2(7.6–23.8) months	6.8 (5.0–9.0) months
	*P*^†^	0.71	0.56
*CDKN2A* mutation	present (*n*= 5)	5.9 (3.3–NA) months	2.9 (1.0–NA) months
	absent (*n* = 50)	16.2 (7.6–23.8) months	7.4 (5.7–10.0) months
	*P*^†^	0.16	**0.015**
*SMAD4* mutation	present (*n*= 5)	13.0 (8.2–NA) months	8.5 (5.9–NA) months
	absent (*n* = 50)	16.2 (7.4–25.4) months	6.8 (4.1–9.0) months
	*P*^†^	0.65	0.90

Annotations:

* median with 95% confidence interval.

† log-rank test.

As compared to samples with wildtype *TP53*, *TP53* mutations were associated with a significantly higher number of additional molecular alterations (Table 3). Cases with *TP53* mutations were significantly younger than cases without this alteration (56.4 ± 11.3 versus 62.8 ± 6.9 years, *P* = 0.04). All five cases aged younger than 45 years and 9 out of 10 cases aged 50 years or younger displayed a *TP53* mutation. Molecular *CDKN2A* alterations were associated with lung metastases: 5 out of 11 cases (46%) with altered *CDKN2A* but only 6 out of 43 cases (14%) with wild-type *CDKN2A* showed lung involvement (*P* = 0.03).

**Table 3:** Number of additional molecular alterations in relation to *TP53* mutational status

	*TP53* mutated (30 cases)		*TP53* unmutated (25 cases)		P^*^
Number of additional mutations (in addition to *TP53*)					0.96
0	13 cases	(43%)	12 cases	(48%)	
1	10 cases	(33%)	10 cases	(40%)	
2	5 cases	(17%)	3 cases	(12%)	
3–4	2 cases	(7%)	0 cases	(0%)	
Mean	0.9 ± 1.0		0.6 ± 0.7		
Number of deletions					0.55
0	21 cases	(70%)	18 cases	(72%)	
1–2	9 cases	(30%)	7 cases	(28%)	
Mean	0.3 ± 0.5		0.3 ± 0.6		
Number of amplifications					0.12
0	18 cases	(60%)	21 cases	(84%)	
1	10 cases	(33%)	4 cases	(16%)	
2–4	2 cases	(7%)	0 cases	(0%)	
Mean	0.7 ± 1.0		0.2 ± 0.4		
Total number of molecular alterations (in addition to *TP53*)					0.03
0	3 cases	(10%)	9 cases	(36%)	
1	14 cases	(47%)	7 cases	(28%)	
2	4 cases	(13%)	5 cases	(20%)	
3	3 cases	(10%)	4 cases	(16%)	
4–5	6 cases	(20%)	0 cases	(0%)	
Mean	1.9 ± 1.4		1.2 ± 1.1		

Annotation:

* Fisher's exact test.

## DISCUSSION

This study comprises a collection of 55 clinically annotated CUP cases with adeno- or undifferentiated carcinoma. By restricting the inclusion criteria to these histologic categories, representing roughly 90% of all CUP patients, we sought to avoid our results to be confounded by the inclusion of rare CUP subgroups. Although no confirmatory conclusions can be expected from this exploratory investigation, our results are suitable to generate hypotheses that might be subject to confirmatory investigations.

The most frequent molecular alterations affected *TP53*, *CDKN2A* and *KRAS*, which were also the top-three alterations in the only other large study of CUP cases [14]. The frequency of *TP53* mutations resembles their frequency in cancer in general [17]. It should be noted that the data on *TP53* alterations in CUP published before the advent of next-generation sequencing were heterogeneous and inconclusive, which is likely explained by small sample sizes and the heterogeneity of both methods and inclusion criteria [10–12].

Overall, the molecular heterogeneity of our cohort does not support the assumption of common biological mechanisms underlying the formation of CUP, instead favoring the notion that CUP is a heterogeneous group of different molecular and clinical entities. A noteworthy correlation was the association of *TP53* mutations with higher numbers of additional molecular alterations, which fits well to the role of *TP53* in maintaining genomic integrity [18, 19], and the correlation of *TP53* mutations with genomic alterations known from other entities, e. g. complex aberrant karyotypes in acute myeloid leukemia [20]. In several tumor entities, *TP53* mutations have been proposed as predictors of poor prognosis [20–22]. In our study on CUP, however, the prognostic impact of *TP53* mutations was less clear, which might be owing to the fact that CUP is a prognostically dismal disease in general and small prognostic differences conferred by *TP53* mutations might have evaded detection. Furthermore, since cancer-associated *TP53* mutations comprise a heterogeneous spectrum of functional defects [23–25], individual mutations might differ from each other with regard to their prognostic impact, and patient populations with different spectra of *TP53* mutations might differ with regard to the role of these mutations as prognostic markers. Interestingly, women not harboring *TP53* mutations constituted a subgroup with relatively favorable prognosis. One might speculate that within this subgroup, gynecological cancers are enriched. Indeed, some gynecological cancers, e.g. cervical cancer, are characterized by relatively low percentages of *TP53*-mutant cases [17], and in several types of gynecological cancers, e.g breast and ovarian cancer, *TP53* mutations are known to confer a poor prognosis [22, 26, 27].

Another interesting finding is the relatively high frequency of *CDKN2A* alterations, being 22% in our cohort and 19% in another study on CUP cases [14]. Frequent *CDKN2A* deletions have been described in some human cancers [28, 29] while the overall frequency of mutations in *CDKN2A* in human cancer has been found to be only 3.8% [17]. It should be mentioned that among the major categories of human tumors, the highest rate of *CDKN2A* mutations has been found in pancreatic cancer [17, 30], and that the mutational spectrum of pancreatic cancer is comparable to the overall spectrum of mutations in CUP detected by us and others [14, 17, 30]. This may either indicate a general biological similarity between these two entities or a frequent origin of CUP from the pancreas, an association that is supported by autopsy studies but weakened by more recent gene expression profiling analyses, which suggest a pancreatic origin of CUP in only 5–12% of cases [31, 32].

The only gene whose mutation was correlated to OS in our cohort was *KRAS*. As for *CDKN2A*, pancreatic cancer comprises the highest percentage of cases with mutant *KRAS* [17]. In addition, *KRAS* mutations have been linked to poor prognosis in this entity [33], again hinting at a possible similarity between pancreatic cancer and CUP. It should be noted that pharmacologic inhibition of the RAS pathway is among the major goals of current anti-cancer drug development, however, drugging mutant RAS itself is not yet feasible [34].

One might define an alteration as druggable when two conditions are met: Firstly, an approved drug has to be available, and secondly, an alteration must be established as a molecular predictor with regard to this drug. Assuming that data on molecular predictors can be transferred from other entities to CUP, 6 of our cases (11%) harbored druggable alterations: 2 cases with *BRAF* V600E mutations, 2 cases with activating *EGFR* mutations, and 2 cases with amplification of *ERBB2*. One might expand this list by the cases with *MET* or *EGFR* amplification, since approved drugs targeting the respective gene products are available. This would elevate the number of potentially druggable patients to 8 cases (15%). Considering the limited therapeutic benefit from currently used standard cytostatic regimens, we conclude that a significant minority of CUP patients may benefit from molecularly stratified therapies. It should be noted that in other defined large entities with comparable frequencies of druggable mutations such as lung cancer, broad spectrum upfront molecular testing is already clinical routine, arguing for comprehensive routine testing in CUP as well. The proportion of druggable cases may rise in the future since drugs targeting additional drivers may soon become available. Examples include FGFR1/3, with specific drugs in late development [35], and the RAS pathway, as already discussed. Clinical trials assessing such molecularly stratified approaches for CUP are urgently needed. In addition, molecular alterations may be useful both as prognostic and predictive markers, e.g. *KRAS* mutations indicating a poor prognosis and, at the same time, predicting lack of response to therapies targeting EGFR, since it is well established that their action requires an intact downstream RAS pathway [34, 36].

We conclude that the vast majority of CUP tumors harbor mutations of relevant driver genes. At least a significant minority of CUP patients are candidates for molecularly stratified therapies, which may contribute to improve the prognosis of this devastating disease.

## MATERIALS AND METHODS

### Patients

Patients were eligible if either adenocarcinoma or undifferentiated carcinoma was histologically confirmed by a board-certified pathologist, and if a primary lesion was not detected despite appropriate search including, as a minimum requirement, cross-sectional imaging of chest and abdomen. 36 cases (Heidelberg cohort) were outpatients seen at the National Center for Tumor Diseases (NCT), Heidelberg, Germany. 19 cases (PACET-CUP cohort) were participants of a German multi-center trial (PACET-CUP study) conducted by the Arbeitsgemeinschaft Internistische Onkologie, Deutsche Krebsgesellschaft (German Cancer Society).

### DNA preparation

After completion of all necessary routine diagnostics, remaining formalin-fixed, paraffin-embedded biopsy specimens were tested for tumor cell content. A tumor cell content of less than 20% and a biopsy size of less than 0.1 cm were considered insufficient for sequencing. Tumor areas were marked on an H&E stained slide. Corresponding tissue areas were microdissected from three subsequent unstained slides. Extraction of genomic DNA was performed after proteinase K digestion and automated purification using the Maxwell 16 Research System (Promega, Madison, USA). DNA content was measured fluorimetrically using the QuBit 2.0 HS DNA Assay (Thermo Fisher Scientific). DNA sequencing grade quality was confirmed using a real-time qPCR-based method (RNAseP Detection system, Thermo Fisher Scientific) [37].

### Library preparation and semiconductor sequencing

For library preparation, the multiplex PCR-based Ion Torrent AmpliSeq^™^ technology (Thermo Fischer Scientific) with the Cancer Hotspot Panel v2 (CHPv2) was used. Amplicon library preparation was performed with the Ion AmpliSeq Library Kit v2.0 using 10 ng of DNA determined by qPCR assay. Briefly, the DNA was mixed with the primer pool, containing all primers for generating the 207 amplicons, and the AmpliSeq HiFi Master Mix in a 20 μl reaction volume and transferred to a PCR cycler (Biometra, Göttingen, Germany). After the end of the PCR, primer end sequences were partially digested using FuPa reagent, followed by ligation of barcoded sequencing adapters (Ion Xpress Barcode Adapters 1–96, Thermo Fisher Scientific). The final library was purified using AMPure XP magnetic beads (Beckman Coulter, Krefeld, Germany) and quantified using qPCR (Ion Library Quantitation Kit, Thermo Fischer Scientific) on a StepOnePlus qPCR machine (Thermo Fischer Scientific). Individual libraries were diluted to a final concentration of 100 pM and eight to ten libraries were pooled and processed to library amplification on Ion Spheres using Ion PGM^™^ Template OT2 200 Kit. Unenriched libraries were quality-controlled using Ion Sphere quality control measurement on a QuBit instrument. After library enrichment (Ion OneTouch ES), the library was processed for sequencing using the Ion Torrent 200bp sequencing v2 chemistry and the barcoded libraries were loaded onto a 318v2 chip.

### Variant calling and annotation

Raw sequencing data were processed using the Ion Torrent Suite Software (version 4.4.3). After base calling, the reads were aligned against the human genome (hg19) using the TMAP algorithm implemented in the Torrent Suite. Variant calling was performed with the variant caller plugin (version 4.4.3) within the Torrent Suite Software using a corresponding bed-file containing the coordinates of the amplified regions. Variant annotation was performed using a custom build variant annotation pipeline in the CLC Genomics Workbench (version 8.0.2). Annotations included information about nucleotide and amino acid changes of RefSeq annotated genes, COSMIC and dbSNP entries as well as detection of possible splice site mutations. For visualization of sequencing and fusion reads, the Integrative Genomic Browser (IGV, http://www.broadinstitute.org/igv/) was used. Only variants with an allele frequency > 5% and minimum coverage > 200 reads were taken into account. For further analysis, only non-synonymous nucleotide exchanges were considered. Each identified variant was compared to entries in the COSMIC, dbSNP and 6500 Exomes databases.

### Copy number variations

Copy number variations (CNVs; amplifications and deletions) were identified by using the coverage data summary for each sample and each amplicon generated by the TorrentSuite software. Detection of CNVs was performed according to Endris et al. [37].

### Statistical analysis

OS and PFS were calculated from the date of histologic confirmation of diagnosis (Heidelberg cohort) or the date of entry into the PACET-CUP study (PACET-CUP cohort). No relevant differences between these two landmark dates are expected because the PACET-CUP study is a trial of first-line therapy. Distribution of survival times was estimated by the method of Kaplan and Meier. The log-rank test was used to test for differences between groups. Cox regression was used to assess prognostic interaction. A two-stage testing procedure starting with the log-rank test [16] was applied to test for differences in the presence of potentially crossing hazards. Median follow-up time was estimated based on time to censoring [38]. Fisher's exact test was used to compare distribution of metastases and alterations between groups. Mann-Whitney test was used to compare age distribution between groups. All *p*-values were two-sided. *P* values below 0.05 were considered statistically significant. Analyses were performed with statistical software R including add-on packages *ComplexHeatmap* and *TSHRC*. If not indicated otherwise, results are summarized as mean ± SD.

## SUPPLEMENTARY MATERIALS FIGURES AND TABLE




