# Supplementary material for: Molecular driver alterations and their clinical relevance in cancer of unknown primary site
Source: Oncotarget. 2016 Jun 14;7(28):44322–9. doi: 10.18632/oncotarget.10035 (PMC5190099; doi:10.18632/oncotarget.10035)
Supplement: Supplementary file 1 [file oncotarget-07-44322-s001.pdf]

Molecular driver alterations and their clinical relevance in cancer of unknown primary site

Supplementary Materials

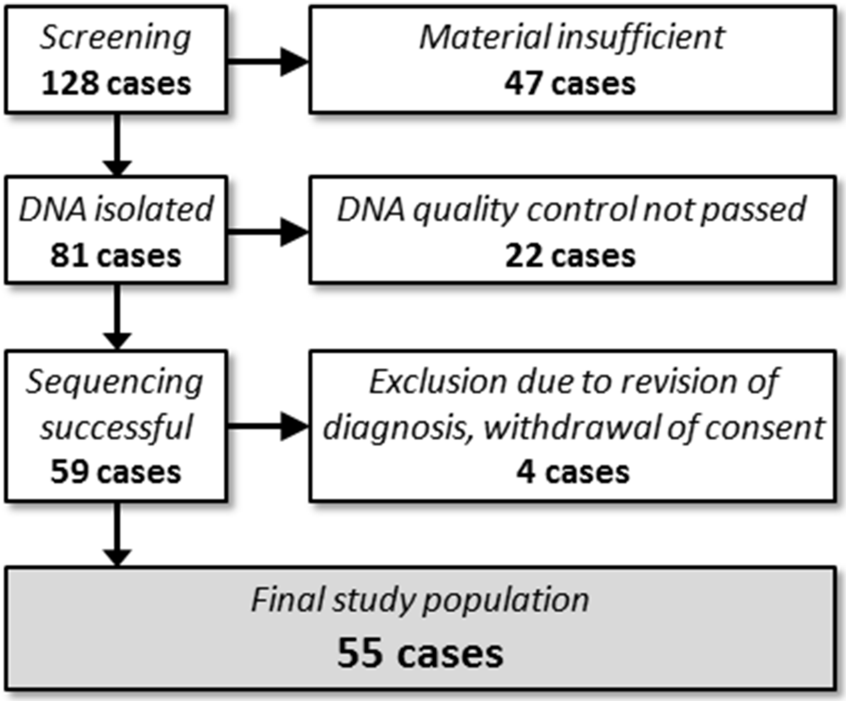

Supplementary Figure S1: Diagram showing the case selection strategy for massive parallel multigene sequencing.

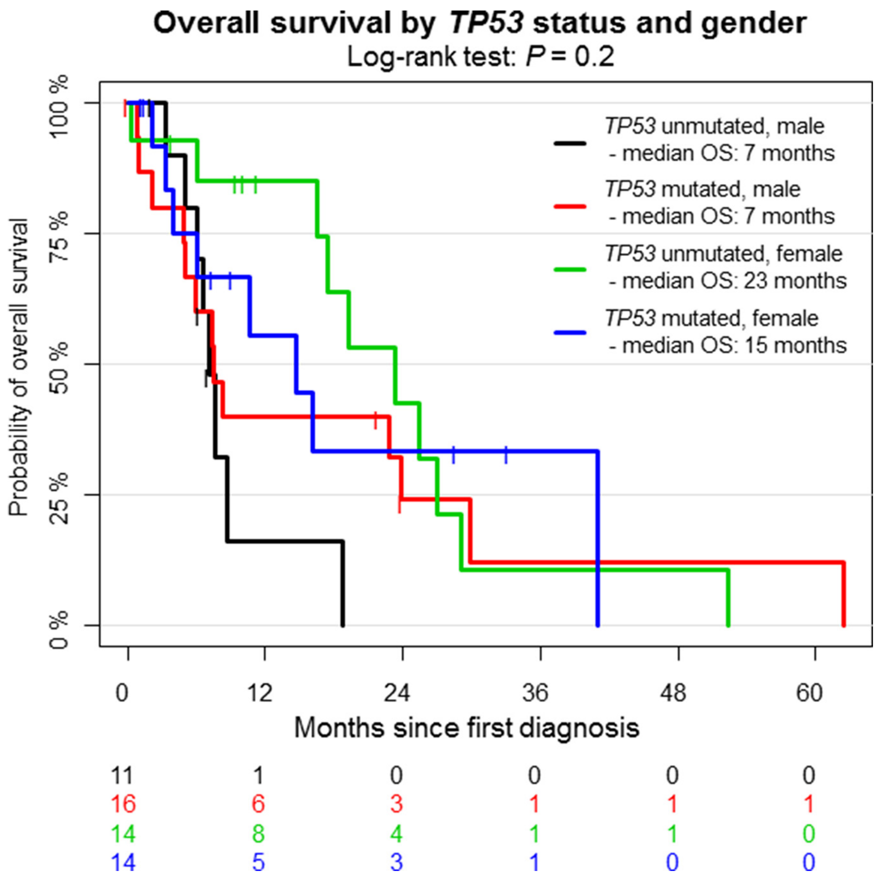

Supplementary Figure S2: Overall survival (OS) of 55 CUP cases depending on TP53 mutational status and gender.

Supplementary Table S1: Panel of genes subjected to sequencing and coverage analysis

ABL1	*EGFR*	*GNAQ*	*KRAS*	*PTPN11*
AKT1	*ERBB2*	*GNAS*	*MET*	*RB1*
ALK	*ERBB4*	*HNF1A*	*MLH1*	*RET*
APC	*EZH2*	*HRAS*	*MPL*	*SMAD4*
ATM	*FBXW7*	*IDH1*	*NOTCH1*	*SMARCB1*
BRAF	*FGFR1*	*IDH2*	*NPM1*	*SMO*
CDH1	*FGFR2*	*JAK2*	*NRAS*	*SRC*
CDKN2A	*FGFR3*	*JAK3*	*PDGFRA*	*STK11*
CSF1R	*FLT3*	*KDR*	*PIK3CA*	*TP53*
CTNNB1	*GNA11*	*KIT*	*PTEN*	*VHL*

Supplementary Table S2: Mutations and CNVs. See Supplementary_Table_S2
