# Supplementary material for: Molecular driver alterations and their clinical relevance in cancer of unknown primary site
Source: Oncotarget. 2016 Jun 14;7(28):44322–9. doi: 10.18632/oncotarget.10035 (PMC5190099; doi:10.18632/oncotarget.10035)
Supplement: Supplementary file 2 [file oncotarget-07-44322-s002.docx]

Supplementary Table S2: Mutations and CNVs

| Sample | Gene | Protein | cDNA | Coverage | Frequency | COSMIC |
| --- | --- | --- | --- | --- | --- | --- |
| 1 | SMAD4 | p.Gly352Arg | NM_005359:c.1054G>A | 1539 | 20% | COSM1150607 |
|  | EGFR | p.Glu746_Ala750delGluLeuArgGluAla | NM_005228:c.2235_2249del | 12497 | 22% | COSM6223 |
|  |  | CNV: amplification |  |  |  |  |
| 2 | KRAS | p.Gly12Phe | NM_004985:c.34_35TT | 2866 | 24% | COSM512 |
|  | Jak2 | CNV: deletion |  |  |  |  |
|  | CDKN2A | CNV: deletion |  |  |  |  |
| 6 | ATM | p.Arg337Cys | NM_000051:c.1009C>T | 2116 | 12% | COSM1350749,COSM21323 |
|  | KRAS | p.Gly12Ala | NM_004985:c.35G>C | 5371 | 15% | COSM1140134,COSM522 |
| 7 | TP53 | p.Glu285fs*21 | NM_000546:c.852dupA | 488 | 43% | no entry |
|  | RB1 | CNV: deletion |  |  |  |  |
| 8 | TP53 | p.Cys135Phe | NM_000546:c.404G>T | 2370 | 48% | COSM303851,COSM303852,COSM2744945,COSM303850,COSM10647,COSM3820723,COSM303849 |
|  | MET | CNV: amplification |  |  |  |  |
| 9 | BRAF | p.Val600Glu | NM_004333:c.1799T>A | 3664 | 27% | COSM476 |
|  | CDKN2A | CNV: deletion |  |  |  |  |
| 10 | TP53 | p.Arg273Leu | NM_000546:c.818G>T | 590 | 24% | COSM318169,COSM3675521,COSM1640828,COSM10779 |
|  | SMAD4 | p.Leu172* | NM_005359:c.515T>A | 790 | 6% | no entry |
| 12 | CDKN2A | p.Glu120* | NM_000077:c.358G>T | 230 | 75% | COSM753749,COSM12479,COSM3092256 |
|  |  | CNV: deletion |  |  |  |  |
|  | TP53 | p.His193Pro | NM_000546:c.578A>C | 2637 | 76% | COSM131461,COSM131458,COSM131459,COSM43833,COSM131460 |
| 13 | TP53 | p.spl? | NM_000546:c.994-1G>T | 2881 | 40% | COSM562343,COSM49007 |
|  |  | p.His179Arg | NM_000546:c.536A>G | 2123 | 19% | COSM1645241,COSM214224,COSM214222,COSM214225,COSM214223,COSM3396280,COSM10889 |
|  | CDKN2A | CNV: deletion |  |  |  |  |
| 14 | RB1 | CNV: deletion |  |  |  |  |
| 15 | TP53 | p.Arg175His | NM_000546:c.524G>A | 2498 | 15% | COSM3355994,COSM1640851,COSM99024,COSM99023,COSM10648,COSM99914,COSM99022 |
| 16 | TP53 | p.Tyr163Cys | NM_000546:c.488A>G | 2053 | 21% | COSM1649381,COSM129854,COSM10808,COSM129855,COSM129852,COSM3388214,COSM129853 |
|  | FGFR3 | CNV: amplification |  |  |  |  |
| 17 | TP53 | p.Tyr205Asp | NM_000546:c.613T>G | 1019 | 22% | COSM1564190,COSM1564188,COSM43844,COSM1564191,COSM1564189 |
|  | NRAS | CNV: amplification |  |  |  |  |
| 18 | MET | CNV: amplification |  |  |  |  |
| 19 | APC | p.Gln1429* | NM_000038:c.4285C>T | 758 | 31% | COSM18836 |
|  | KRAS | p.Gly12Cys | NM_004985:c.34G>T | 1312 | 26% | COSM1140136,COSM516 |
|  | TP53 | p.Arg267Trp | NM_000546:c.799C>T | 424 | 39% | COSM1640829,COSM11183,COSM3717629,COSM179804 |
| 20 | KRAS | p.Gly12Cys | NM_004985:c.34G>T | 2103 | 38% | COSM1140136,COSM516 |
|  | CDKN2A | CNV: deletion |  |  |  |  |
| 21 | EGFR | p.Gly719Cys | NM_005228:c.2155G>T | 1844 | 30% | COSM6253 |
|  | CDKN2A | CNV: deletion |  |  |  |  |
| 22 | BRAF | p.Val600Glu | NM_004333:c.1799T>A | 2001 | 45% | COSM476 |
|  | EGFR | CNV: low level amplification or polysomy |  |  |  |  |
| 23 | APC | p.Arg1450* | NM_000038:c.4348C>T | 820 | 4% | COSM13127 |
|  | TP53 | p.Arg273His | NM_000546:c.818G>A | 814 | 38% | COSM99729,COSM1645335,COSM3356963,COSM10660 |
|  | FGFR3 | CNV: low level amplification or polysomy |  |  |  |  |
|  | APC | CNV: deletion |  |  |  |  |
|  | HNF1A | CNV: amplification |  |  |  |  |
| 24 | ATM | p.Asp2725Asn | NM_000051:c.8173G>A | 1340 | 16% | no entry |
| 25 | KRAS | p.Gly12Ala | NM_004985:c.35G>C | 1246 | 52% | COSM1140134,COSM522 |
|  | TP53 | p.Pro82fs*70 | NM_000546:c.242_243insAGCTCCTACA | 1053 | 31% | no entry |
|  | SMAD4 | p.Tyr260fs*76 | NM_005359:c.777delT | 1112 | 29% | no entry |
|  | GNA11 | CNV: amplification |  |  |  |  |
| 26a | TP53 | p.Glu336* | NM_000546:c.1006G>T | 1024 | 70% | COSM11291,COSM1522200 |
|  | ERBB2 | p.Leu755Pro | NM_004448:c.2263_2264CC | 4096 | 63% | COSM683 |
|  |  | CNV: amplification |  |  |  |  |
|  | CSF1R | CNV: amplification |  |  |  |  |
|  | RB1 | CNV: deletion |  |  |  |  |
|  | AKT1 | CNV: amplification |  |  |  |  |
| 27 | SMAD4 | p.Ser504Arg | NM_005359:c.1512T>G | 2216 | 12% | no entry |
| 28 | CDKN2A | CNV: deletion |  |  |  |  |
| 29 | TP53 | p.Tyr220Ser | NM_000546:c.659A>C | 1849 | 68% | COSM43850,COSM3675522,COSM3675523,COSM251428,COSM251426,COSM251427 |
|  | NRAS | CNV: amplification |  |  |  |  |
|  | FGFR1 | CNV: amplification |  |  |  |  |
|  | GNA11 | CNV: amplification |  |  |  |  |
|  | JAK3 | CNV: amplification |  |  |  |  |
| 30 | STK11 | p.Phe354Leu | NM_000455:c.1062C>G | 800 | 58% | COSM21360 |
| 31 | ERBB4 | p.Leu175Val | NM_005235:c.523T>G | 6028 | 14% | no entry |
|  | CDKN2A | p.Arg58* | NM_000077:c.172C>T | 2758 | 36% | COSM1624870,COSM12473,COSM99731,COSM99730 |
|  | CDH1 | p.Val77fs*17 | NM_004360:c.226dupA | 5520 | 15% | no entry |
|  | TP53 | p.Gly245Ser | NM_000546:c.733G>A | 4697 | 12% | COSM1640833,COSM121036,COSM6932,COSM121035,COSM121037,COSM3356965 |
|  | FGFR3 | CNV: deletion |  |  |  |  |
| 32 | FGFR1 | p.Gly268Cys | NM_015850:c.802G>T | 1099 | 7% | no entry |
|  | TP53 | p.Arg181Cys | NM_000546:c.541C>T | 1461 | 17% | COSM3712584,COSM131465,COSM131462,COSM1638400,COSM131463,COSM11090,COSM131464 |
|  | TP53 | p.Arg110His | NM_000546:c.329G>A | 754 | 53% | COSM46115 |
|  | ERBB2 | p.Leu755Ser | NM_004448:c.2264T>C | 1587 | 16% | COSM14060 |
| 33 | KRAS | p.Gly12Leu | NM_004985:c.34_35CT | 4793 | 41% | COSM514 |
|  | TP53 | p.Arg342fs*3 | NM_000546:c.1024delC | 5332 | 42% | COSM128665,COSM18597,COSM45639 |
|  | SMAD4 | p.Glu394* | NM_005359:c.1180G>T | 790 | 32% | no entry |
| 35 | TP53 | p.Arg249Thr | NM_000546:c.746G>C | 2165 | 10% | COSM1728798,COSM375643,COSM43665,COSM3403260,COSM375642 |
|  | FGFR3 | CNV: deletion |  |  |  |  |
| 37 | CDKN2A | p.Trp110* | NM_000077:c.329G>A | 401 | 27% | COSM12481,COSM126617,COSM126618 |
| 40 | TP53 | p.Arg175Pro | NM_000546:c.524G>C | 1436 | 18% | COSM45416 |
|  |  | p.Tyr163Cys | NM_000546:c.488A>G | 1456 | 31% | COSM1649381,COSM129854,COSM10808,COSM129855,COSM129852,COSM3388214,COSM129853 |
|  | NRAS | CNV: amplification |  |  |  |  |
| 41 | PIK3CA | p.Ser66Tyr | NM_006218:c.197C>A | 996 | 29% | no entry |
|  | TP53 | p.Gln167fs*4 | NM_000546:c.498_499insAA | 2637 | 22% | no entry |
| 42 | APC | p.Arg1450* | NM_000038:c.4348C>T | 1538 | 19% | COSM13127 |
|  | CDKN2A | p.Arg58* | NM_000077:c.172C>T | 1860 | 25% | COSM1624870,COSM12473,COSM99731,COSM99730 |
|  | KRAS | p.Gly12Asp | NM_004985:c.35G>A | 2220 | 17% | COSM521,COSM1135366 |
|  | TP53 | p.Arg175His | NM_000546:c.524G>A | 2189 | 25% | COSM3355994,COSM1640851,COSM99024,COSM99023,COSM10648,COSM99914,COSM99022 |
|  | GNAS | p.Arg201His | NM_000516:c.602G>A | 887 | 17% | COSM94388,COSM27895 |
| 43 | VHL | p.Lys171Asn | NM_000551:c.513G>T | 508 | 51% | COSM14374 |
|  | TP53 | p.Cys176Phe | NM_000546:c.527G>T | 690 | 38% | COSM10645,COSM117395,COSM117396,COSM117397,COSM117398,COSM1640850 |
| 44 | TP53 | p.Gly154fs*16 | NM_000546:c.461delG | 1574 | 30% | COSM5315968 |
| 45 | TP53 | p.Glu294fs*51 | NM_000546:c.880delG | 927 | 8% | COSM318363,COSM6621 |
| 46 | PIK3CA | p.Glu110del | NM_006218:c.325_327delGAA | 269 | 35% | COSM24710,COSM445994 |
|  | PTEN | p.Arg130Gln | NM_000314:c.389G>A | 5688 | 63% | COSM5033 |
|  |  | CNV: amplification |  |  |  |  |
| 47 | ATM | p.Val1941Leu | NM_000051:c.5821G>C | 363 | 30% | COSM21922 |
|  | KRAS | p.Gly13Asp | NM_004985:c.38G>A | 1397 | 27% | COSM1140132,COSM532 |
|  | TP53 | p.Gly245Ser | NM_000546:c.733G>A | 3633 | 36% | COSM1640833,COSM121036,COSM6932,COSM121035,COSM121037,COSM3356965 |
|  | ALK | CNV: amplification |  |  |  |  |
|  | AKT1 | CNV: low level amplification or polysomy |  |  |  |  |
| 48 | PTEN | p.Gln17* | NM_000314:c.49C>T | 1782 | 20% | COSM5153 |
|  | TP53 | p.Arg213* | NM_000546:c.637C>T | 1000 | 15% | COSM99618,COSM1638393,COSM99616,COSM10654,COSM99617,COSM3378350,COSM99615 |
|  | FGFR3 | CNV: low level amplification or polysomy |  |  |  |  |
| 49 | TP53 | p.Arg273Pro | NM_000546:c.818G>C | 1363 | 72% | COSM43896,COSM1646808,COSM165077 |
|  | CDKN2A | CNV: deletion |  |  |  |  |
| 51 | TP53 | p.Arg306* | NM_000546:c.916C>T | 1101 | 9% | COSM3388168,COSM10663,COSM1640820,COSM99947 |
|  | KRAS | CNV: amplification |  |  |  |  |
| 52 | KRAS | p.Gly12Asp | NM_004985:c.35G>A | 2223 | 32% | COSM521,COSM1135366 |
|  | TP53 | p.Gly245Ser | NM_000546:c.733G>A | 5347 | 33% | COSM1640833,COSM121036,COSM6932,COSM121035,COSM121037,COSM3356965 |
| 53 | CDKN2A | p.Arg58* | NM_000077:c.172C>T | 913 | 38% | COSM1624870,COSM12473,COSM99731,COSM99730 |
|  | TP53 | p.Arg273Cys | NM_000546:c.817C>T | 397 | 28% | COSM3355991,COSM10659,COSM1645518,COSM99933 |
|  | RB1 | CNV: deletion |  |  |  |  |
|  | AKT1 | CNV: amplification |  |  |  |  |
| 54 | BRAF | p.Gly466Ala | NM_004333:c.1397G>C | 332 | 37% | COSM452 |
|  | ERBB2 | CNV: amplification |  |  |  |  |
|  | SMAD4 | CNV: deletion |  |  |  |  |
| 55 | ATM | p.Asp841Asn | NM_000051:c.G2521A | 3548 | 62% | no entry |
|  | TP53 | p.Tyr220Cys | NM_000546:c.659A>G | 1393 | 94% | COSM99718,COSM3355993,COSM99719,COSM1644277,COSM99720,COSM10758 |
